# Rigidity of the Outer Shell Predicted by a Protein Intrinsic Disorder Model Sheds Light on the COVID-19 (Wuhan-2019-nCoV) Infectivity

**DOI:** 10.3390/biom10020331

**Published:** 2020-02-19

**Authors:** Gerard Kian-Meng Goh, A. Keith Dunker, James A. Foster, Vladimir N. Uversky

**Affiliations:** 1 Goh’s BioComputing, Singapore 548957, Singapore; 2 Center for Computational Biology, Indiana and Bioinformatics, Indiana University School of Medicine, Indianapolis, IN 46202, USA; kedunker@iupui.edu; 3 Department of Biological Sciences, University of Idaho, Moscow, ID 83844, USA; foster@uidaho.edu; 4 Institute for Bioinformatics and Evolutionary Studies, University of Idaho, Moscow, ID 83844, USA; 5 Department of Molecular Medicine, Morsani College of Medicine, University of South Florida, Tampa, FL 33612, USA; 6 Institute for Biological Instrumentation of the Russian Academy of Sciences, Federal Research Center ‘Pushchino Scientific Center for Biological Research of the Russian Academy of Sciences’, Moscow region, 142290 Pushchino, Russia

**Keywords:** COVID-19, Wuhan-2019-nCoV, protein intrinsic disorder, shell disorder, virulence, transmission, nucleocapsid protein, membrane protein

## Abstract

The world is currently witnessing an outbreak of a new coronavirus spreading quickly across China and affecting at least 24 other countries. With almost 65,000 infected, a worldwide death toll of at least 1370 (as of 14 February 2020), and with the potential to affect up to two-thirds of the world population, COVID-19 is considered by the World Health Organization (WHO) to be a global health emergency. The speed of spread and infectivity of COVID-19 (also known as Wuhan-2019-nCoV) are dramatically exceeding those of the Middle East respiratory syndrome coronavirus (MERS-CoV) and severe acute respiratory syndrome coronavirus (SARS-CoV). In fact, since September 2012, the WHO has been notified of 2494 laboratory-confirmed cases of infection with MERS-CoV, whereas the 2002–2003 epidemic of SARS affected 26 countries and resulted in more than 8000 cases. Therefore, although SARS, MERS, and COVID-19 are all the result of coronaviral infections, the causes of the coronaviruses differ dramatically in their transmissibility. It is likely that these differences in infectivity of coronaviruses can be attributed to the differences in the rigidity of their shells which can be evaluated using computational tools for predicting intrinsic disorder predisposition of the corresponding viral proteins.

Before the MERS-CoV outbreak in 2012, we built a model using artificial intelligence (AI) tools for the identification of the levels of predicted intrinsic disorder (PID) in a query protein using its amino acid sequence as the only input [1]. Next, this information was applied to rank various coronaviruses based on the levels of disorder in their inner and outer shells (i.e., PID values evaluated for their nucleocapsid (N) and membrane (M) proteins, respectively) and to find if these shell PID values were indicative of how long the given virus was able to stay in the environment [1,2,3]. The application of this model ranked coronaviruses into three groups (Categories A, B, and C), members of which differ not only by the levels of their shell disorder, but also by their respiratory and fecal-oral transmission potential [1,2,3]. The model was consistent with the hypothesis that viruses remaining in harsh environments require harder, i.e., less disordered, shells to survive [2,3]. Furthermore, higher levels of inner shell disorder could be associated with greater infectivity, especially with regard to the viruses with high respiratory transmission potential [3,4,5]. 

On the basis of these considerations, Category A coronaviruses are characterized by more disordered inner (N) shell and higher levels of respiratory transmission combined with lower levels of fecal-oral transmission. Category B coronaviruses are characterized by the N shell having intermediate levels of disorder and intermediate levels of respiratory and fecal-oral transmission, whereas Category C coronaviruses possess the most ordered N proteins and are characterized by higher levels of fecal-oral transmission and lower levels of respiratory transmission [1,2,3,4,5]. Notably, some (not all) of the viruses with lower outer shell rigidity (higher M PID) are in Category C with greater fecal-oral transmission potential because these viruses do not have to be in the environment for a long time given that the fecal-oral route is an efficient mode of transmission among some farm animals.

According to this PID-based classification, SARS-CoV (with PIDs of 9% and 50% for its M and N proteins, respectively) falls into Category B, which includes the coronaviruses with the intermediate levels of both respiratory and fecal-oral potential. However, MERS-CoV, whose M and N proteins are characterized by the PID values of 10% and 44%, respectively, were placed into a group with a higher fecal-oral and lower respiratory transmission potential, Category C [5]. Importantly, this classification of coronaviruses which is based on the rigidity of their shells was generally supported by clinical observations of their transmission modes. 

The usefulness of our model is now becoming obvious, considering the current COVID-19/Wuhan 2019-nCoV outbreak [6]. Currently, the analysis suggests (details of this analysis will be published elsewhere [7]) that COVID-19/Wuhan 2019-nCoV belongs to Category B, indicating that, similar to SARS-CoV, COVID-19/Wuhan 2019-nCoV can be efficiently transmitted via the respiratory mode. However, due to the fact that the outer shell of this virus is among the hardest in the coronavirus family, the model suggests that COVID-19/Wuhan 2019-nCoV is likely to be more resilient in body fluids and the environment than most of its cousins, including SARS-CoV and MERS-CoV. 

Therefore, our model predicts that COVID-19/Wuhan 2019-nCoV can be efficiently spread by respiratory means, as in the case of SARS-CoV, but it also has the potential to remain outside the body longer than SARS-CoV, and, probably, MERS-CoV. The possibility of fecal-respiratory transmission of COVID-19/Wuhan 2019-nCoV is especially of concern, as was the case for SARS-CoV in the Amoy Garden, Hong Kong3. Furthermore, the intermediate levels of fecal-oral transmission potential of COVID-19/Wuhan 2019-nCoV should also be kept in mind, as in the case of all other viruses in Category B. 

Although the ability to last longer outside the body provides better fitness of the virus that relies more on the fecal-respiratory route of transmission, as seen in PEDV3,5, transmission of the more rigid virus can occur via feces, but also in airborne form released from mucus, vomit, and other body fluids. These properties can also explain the higher contagiousness levels of the COVID-19/Wuhan 2019-nCoV than with SARS-CoV [6]. In fact, the ability of COVID-19/Wuhan 2019-nCoV to remain infectious outside the body for a longer period than SARS-CoV could imply that it requires fewer viral particles for greater chances of infection. Furthermore, an infected body can shed more active viral particles in greater concentrations as more particles are able to resist the digestive enzymes found in most bodily fluids including saliva [2,3]. All these can explain not only high contagiousness of the COVID-19/Wuhan 2019-nCoV but also the reported capability of this virus to be spread even before the patient begins to show symptoms [6].
